# Confronting Inequalities and Bridging the Divide: A Retrospective Study Assessment of Country-Level COVID-19 Vaccine Equality with a Cox Regression Model

**DOI:** 10.3390/vaccines12050552

**Published:** 2024-05-18

**Authors:** Lan Cheng, W. K. Chan, Lijie Zhu, Melody H. Chao, Yang Wang

**Affiliations:** 1 Big Data Bio-Intelligence Laboratory, Big Data Institute, The Hong Kong University of Science and Technology, Hong Kong, China; yangwang@ust.hk; 2 Department of Computer Science and Engineering, The Chinese University of Hong Kong, Hong Kong, China; wkinchan@outlook.com; 3 Faculty of Medicine, The Chinese University of Hong Kong, Hong Kong, China; zhulijiecuhk@outlook.com; 4 College of Art and Design, Shenzhen University, Shenzhen 518060, China; chao@szu.edu.cn

**Keywords:** COVID-19 pandemic, vaccine equity, human development index, global health system

## Abstract

COVID-19 vaccination is vital in reducing illness, hospitalization, and mortality in the face of this global pandemic. However, COVID-19 vaccination rates worldwide remain below WHO public health targets, and persistent structural inequities reduce vaccine uptake likelihood among populations of low socioeconomic status. We conducted a cross-sectional study based on publicly available data from the Our World in Data project. We included all 124 countries with available open epidemic data and a population of more than 5 million. We used a Cox Regression Model, with population, population density, median age, human development index, GDP per capita, gender inequality index, healthcare access and quality index, hospital beds per thousand people, completion rate of primary education, infection cases of COVID-19 by the end of 2022, and death rate due to COVID-19 by the end of 2022 as predictors for model hazard rates of completion of 50% population vaccination. According to our study, countries with higher populations, higher population density, higher human development index, lower gender inequality index, and lower hospital beds per 1000 people had a higher hazard rate, which means they were more likely to achieve 50% population vaccination faster. By utilizing the time to achieve vaccination rate goals as our primary endpoint, we evaluated inequity from a dual perspective, considering both the differences in vaccination rates and the duration required to attain them. Consequently, this study employed survival analysis approaches to gain a comprehensive understanding of vaccine drivers and population-level trends nationally and inform all communities from a statistical perspective to prepare for health emergencies. Development-level standing modified the effects of equal access to COVID-19 vaccination on cumulative cases and mortality, for which countries of low or medium human development tended to fare worse in outcomes than high human development countries. As COVID-19 vaccination efforts evolve, healthcare professionals, scholars, and policymakers need to identify the structural impediments to equitable vaccination awareness, access, and uptake so that future vaccination campaigns are not impeded by these barriers to immunization. Recognizing the complex nature of this significant barrier, it is evident that no single statistical analysis method can comprehensively address all intricacies.

## 1. Introduction

The global burden of infectious and non-communicable diseases is unevenly distributed across different countries, primarily due to disparities in access to vaccines and medicines. The COVID-19 pandemic, which resulted in millions of cases and deaths worldwide, further highlighted the challenges in distributing and ensuring the uptake of vaccines and medicines. To address the unequal distribution of diseases and reduce global inequality, it is necessary to reform the health and social systems of all countries, particularly High-Income Countries (HICs), Low-Income Countries (LICs), and Low-to-Middle Income Countries (LMICs).

The post-pandemic period presents an opportunity to proactively anticipate and address potential future public health emergencies at both macro and micro levels. Governments should prioritize strengthening the resilience of their health systems by enhancing prevention and response capacities, improving preparedness strategies, and bolstering primary care services. These initiatives can establish a more robust and effective healthcare infrastructure, capable of managing and mitigating the impact of future crises.

However, accurately quantifying the impact of COVID-19 vaccines in terms of disease burden reduction and mortality poses challenges. There is a lack of published studies investigating country-level COVID-19 vaccine equality and its potential determinants. To fill this knowledge gap, this paper presents a comprehensive analysis of country-level inequality in COVID-19 vaccination rates using the survival analysis method. The findings contribute to understanding equity gaps in global vaccination efforts and provide insights for targeted interventions to ensure inclusive and equitable access to COVID-19 vaccines. Addressing and mitigating inequalities is crucial for effectively combating the COVID-19 pandemic on a global scale.

### 1.1. Background

The COVID-19 pandemic has had a profound global impact, presenting significant challenges across various aspects of people’s lives. The development, testing, and distribution of COVID-19 vaccines were crucial in mitigating the disease’s morbidity and mortality rates. The World Health Organization (WHO) set a goal of achieving 70% global vaccination coverage by mid-2022. Achieving this target requires sustained efforts, collaboration, and national leadership to implement vaccination strategies tailored to each country’s specific needs. To support global vaccination targets, it is crucial to prioritize vaccinating high-risk populations, including the elderly, individuals in limited healthcare systems, and those with co-morbidities or immunocompromised conditions. Focusing on fully vaccinating these vulnerable groups can yield the greatest benefits in terms of public health and reducing severe outcomes. Despite recognizing the adverse outcomes associated with vaccine inequity, there remains a substantial disparity in the distribution of COVID-19 vaccines [1,2,3,4,5]. High-Income Countries (HICs) have significantly greater access to vaccines compared to Low–Middle-Income Countries (LMICs), resulting in disproportionately high rates of COVID-19 infections and fatalities in LMICs. The negative consequences of global vaccine inequity have been widely acknowledged, and there is a growing consensus that equal access to opportunities should be afforded to all individuals [3].

To address aggravating inequity brought about by the global pandemic challenges on the domestic side, continued efforts have been made to tackle systemic disparities and ensure equitable access to healthcare services, including testing, treatment, and vaccination. On the global side, various initiatives have been launched to address the inequality challenge. For instance, the COVAX facility aims to negotiate affordable vaccine prices and ensure equitable access for LMICs. Led by Gavi, the Vaccine Alliance, the COVAX program serves as the vaccine component of the Access to COVID-19 Tools (ACT) Accelerator to ensure global equitable access to COVID-19 vaccines [6]. More precisely, addressing these structural inequities requires a comprehensive approach that involves policy changes, increased investment in underserved communities, culturally competent care, and efforts to reduce systemic biases and discrimination within healthcare systems. 

Current vaccine efforts have highlighted significant disparities in the distribution of COVID-19 vaccines, with high-income countries having better access compared to low- and middle-income countries. Vulnerable populations, including the elderly and marginalized communities, often face barriers to vaccine access. Socioeconomic and racial/ethnic disparities, along with vaccine hesitancy and limited healthcare infrastructure, contribute to inequitable vaccine distribution. Addressing these issues requires prioritizing vulnerable populations, building trust, improving access to healthcare infrastructure, and fostering international collaboration [5,6,7,8,9,10]. The global impact of the COVID-19 pandemic has exposed the vulnerabilities and inadequate preparedness of health systems worldwide. Many Low–Middle-Income Countries (LMICs) face significant challenges preserving lives and maintaining essential healthcare services. This highlighted the strain on healthcare systems and the need for improved resilience and capacity to respond to future crises effectively.

### 1.2. Literature Review

The COVID-19 pandemic has highlighted the vulnerability of countries worldwide and their abilities to cope with the emergent demands of medical support amid global crises [9,10]. The literature on COVID-19 vaccination rates and equity has demonstrated the significant disparities in vaccine distribution, access, and uptake across different countries and populations [3,11,12,13]. The existing literature has extensively examined the influence of various country-level factors on racial and ethnic disparities in influenza immunization rates globally. These factors include GDP level, vaccine accessibility, education level, income, employment status, housing segregation, and rurality [1,6,11,14]. 

Grounded in previous research, systemic or structural inequities within health systems refer to the underlying and often deep-rooted factors that perpetuate disparities in healthcare access, quality, and outcomes among different racial and ethnic groups. These inequities are not isolated incidents but result from a complex interplay of social, economic, and political factors that shape the healthcare landscape [7,8,9]. Studies have shown that Low-Income Countries and marginalized populations often face challenges in accessing vaccines, leading to inequitable distribution. Factors such as limited healthcare infrastructure, vaccine hesitancy, and geopolitical considerations contribute to these disparities [5,10,15].

Survival analysis, specifically the Cox regression model, has been utilized in previous studies to analyze vaccine distribution and equity. For instance, a study employed survival analysis to assess the time required for countries to achieve specific vaccination rate goals. The study found that countries with higher healthcare resources and stronger public health systems tended to achieve their vaccination goals more rapidly, indicating a potential inequality in vaccine distribution. Other studies utilized survival analysis to explore the impact of socioeconomic factors and healthcare infrastructure on the time to reach vaccination goals. The results revealed that countries with stronger healthcare systems and higher socioeconomic status achieved vaccination targets more rapidly, highlighting the importance of addressing systemic inequalities [13,16,17,18].

However, a gap in the literature remains regarding a comprehensive evaluation of vaccine equity that considers both vaccination rates and the temporal aspect of achieving these rates. This is an area to which the current study aims to contribute. The study will assess country-level COVID-19 vaccine equality from a two-dimensional perspective using the Cox regression model within a retrospective framework. More specifically, our study will examine the differences in vaccination rates among countries and the time duration required for each country to reach well-recognized vaccination rate targets. By incorporating time as a critical variable, the study aims to provide a more nuanced understanding of vaccine distribution and access inequities. Additionally, the study aims to address the gap in the literature by exploring the potential impact of various factors on observed vaccine inequalities [17,19]. By analyzing the relationship between these factors and vaccine equity, our study seeks to provide insights that can inform strategies to improve equitable vaccine distribution.

## 2. Statement of Contribution

### 2.1. Challenges Regarding Inequality in Vaccination Rates among Countries

Vaccination disparities have significant negative impacts on marginalized groups. Still, some factors can be addressed and rectified to promote more significant equity within healthcare systems. Documented studies have investigated countries with smaller economies that experienced a significant delay in initiating their vaccination campaigns compared to larger economies, with differences of up to 100 days. Among low-income countries specifically, for each additional day it took to administer the first vaccine dose, there was a 1.92% increase in cumulative COVID-19 cases compared to high-income countries after accounting for factors such as population size, median age, and availability of testing data (*p* = 0.0395). These findings underscore the importance of timely vaccine distribution and highlight the potential consequences of delayed vaccination efforts on the spread and impact of the virus [1,2,3,5]. One aspect of structural inequities is unequal access to medical interventions or healthcare professionals. Marginalized groups, including racial and ethnic minorities, often face barriers to accessing healthcare providers due to various factors, such as geographic location, socioeconomic status, language barriers, and discrimination. The limited availability of healthcare professionals in certain areas and disparities in the distribution of healthcare resources further exacerbate these inequities. Another component of structural inequities is the issue of inadequate payment for care. Financial barriers, including lack of health insurance coverage or underinsurance, can prevent individuals from seeking timely and necessary healthcare services. This can result in delayed or deferred care, leading to worsened health outcomes and disproportionate burdens on marginalized populations. 

### 2.2. Comprehensive Assessments to Identify and Address Disparities in COVID-19 Vaccine Access

The persistence of national disparities in access to COVID-19 vaccination can be attributed to various factors, such as insufficient vaccine education advocacy, unequal distribution of healthcare facilities and providers, limited availability of culturally competent care, and implicit biases that influence provider–patient interactions. Marginalized populations may face challenges in finding healthcare professionals who understand their specific cultural, linguistic, and social needs, leading to disparities in access to appropriate and quality care for vaccination. In addition, inadequate payment for care is another structural inequity contributing to disparities. Marginalized groups in LMICs often experience financial barriers to healthcare, including limited health insurance coverage and high out-of-pocket costs [2,3,9,10,11]. Recognizing and addressing these structural inequities from a statistical perspective is crucial for promoting health equity and improving the health outcomes of marginalized regional groups. By understanding that these disparities are not simply individual shortcomings but are rooted in systemic factors, health systems can work towards implementing measures that ensure equitable access to vaccines for the nextfuture health emergencies, eliminate financial barriers, and improve healthcare outcomes for all populations, regardless of race or ethnicity.

### 2.3. Objectives of the Study

A sustainable push by countries to implement their vaccination strategies, tailored to their unique contexts and populations, will contribute to achieving the global vaccination coverage target. This requires a combination of factors, including an integrated healthcare system, sufficient vaccine supply, efficient distribution systems, robust outreach campaigns, and equitable vaccine access for all eligible individuals. Therefore, obtaining a better data-driven understanding of the role of global vaccine equity is essential.

Vaccination rates show pattern of early disparities that became entrenched. By the end of 2022, Burundi had the lowest vaccination rate among all countries, with only 0.23 percent of the population having received at least one dose of a vaccine. In contrast, the United Arab Emirates achieved a comprehensive vaccination rate, with 100 percent of its population receiving at least one vaccine dose. In view of the disparities discussed among countries, our study aims to achieve the following objectives.

First, to quantify and compare the time taken for the first vaccine rollout by human development index category using the available data.

Second, to quantify and compare the time taken for vaccinating 50% of the population by human development index category using publicly accessible data.

Third, to investigate whether other variables, including population, median age, healthcare resources, education availability, and COVID-19 epidemic severity, affect the time to achieve 50% population vaccination.

## 3. Materials and Methods

While the World Health Organization (WHO) aimed to achieve a 70% vaccination rate by the end of 2022, the current reality falls considerably short of that target. To ensure an adequate number of events for the model, this study utilizes a 50% vaccination rate as the designated event threshold. This adjusted threshold allows for a more feasible and meaningful analysis, considering the available data and the progress made in vaccination efforts thus far. By prioritizing the vaccination of high-risk populations and adopting country-led approaches, countries can make significant progress in fulfilling their national goals and the global target of reaching 70% vaccination coverage. This collective effort will contribute to reducing the negative impact of the COVID-19 pandemic, protecting vulnerable populations, and ultimately achieving a higher level of population immunity worldwide for future global epidemic disasters. By leveraging online platform data from 124 countries with a population exceeding 5 million, this study provides valuable insights into the temporal dynamics and disparities in vaccine distribution, uptake, and delivery.

### 3.1. Study Design and Data Sources

This study utilized a retrospective study design to examine variations in the timing of COVID-19 vaccine uptake and its association with various country-level characteristics. We used two endpoints: time to first vaccination and time to 50% vaccination rate. The time taken for the first vaccination in each country was determined by calculating the number of days between the date of the first vaccination reported by the World Health Organization (WHO) for each country and the global date of the first-ever vaccination (3 December 2020). The time taken for the 50% vaccination rate was calculated with the same start date, while the end date became the day half the country’s population was vaccinated. This study considers three factors regarding the decision to use a 50% vaccination rate as the event threshold.

(1) Realistic Attainability: The decision to adjust the event threshold to a 50% vaccination rate considers the current progress and challenges in global vaccination campaigns. Therefore, selecting a lower 50% vaccination rate threshold reflects a more realistic and attainable milestone within the given timeframe. (2) Sufficient Events for Analysis: Using a 50% vaccination rate as the event threshold ensures that there will be a reasonable number of events available for analysis. The lower threshold allows for a larger sample size of countries that have reached or surpassed the 50% vaccination rate, enhancing the robustness and reliability of the analysis. (3) Monitoring Progress and Outcomes: Setting a 50% vaccination rate as the event threshold provides a valuable benchmark for tracking the progress and outcomes of vaccination efforts. It allows for evaluating countries’ performance in reaching the halfway mark regarding vaccine coverage, which can provide insights into the effectiveness of vaccination strategies and their impact on COVID-19 outcomes. Each country served as the unit of analysis in this study. In this context, the data used in this analysis is sourced from governmental health authorities and compiled by the Our World in Data project (an international vaccination dataset publicly accessible worldwide) at the University of Oxford, as detailed in Table 1. 

### 3.2. Selection Criteria for Study Countries

We selected 124 countries with open COVID-19 data in the Our World in Data project [20] and with a population of more than 5 million. Since countries with too small a population may be more likely to have an unpredictable vaccination rate, we removed these countries from our model. Table 2 provides a summary of the population sizes and characteristics of the included countries.

### 3.3. Data Analysis

We performed a retrospective study assessing country-level vaccination equity using a Cox regression model of survival analysis, in which (1) population, (2) population density, and (3) a country’s economic standing were assessed using the United Nations Development Programme (UNDP) compilation of the Human Development Index (HDI) for 191 countries. The HDI index assesses the level of human development in a country by taking into account factors such as health, education, income, and living conditions. It offers a comparative measure of human development across countries and over time, including (4) gross domestic product based on the purchasing power parity (GDP PPP) per capita category; (5) gender inequality index; (6) healthcare access and quality index; (7) hospital beds per thousand people; (8) completion rate of primary education; (9) infection cases of COVID-19 by the end of 2022; (10) death rate due to COVID-19 by the end of 2022 were used as predictors, and the endpoint of this model was the time from the world first COVID-19 vaccination to 50% population vaccinated by the end of 2022 for each country. For countries that did not have 50% vaccination, 31 December 2022 was used as the censoring date in the model. The SAS procedure PROC PHREG was used to build the Cox regression model and calculate related estimates. We conducted a stepwise regression analysis to select the significant variables from those variables of interest in the model. A variable has to be significant at the 0.25 level before it can be entered into the model, while a variable in the model has to be significant at the 0.15 level for it to remain in the model.

Survival analysis, as an epidemiological methodology that employs time to event as the primary endpoint, enables a comprehensive examination of a given phenomenon’s temporal progression and outcomes [21,22,23]. In assessing vaccination rates, survival analysis provides a means to discern whether the event of achieving a 50% vaccination rate occurs and the temporal dynamics associated with its realization. This analytical approach facilitates a more nuanced understanding of the multifaceted inequalities that exist among countries. Specifically, it recognizes that even when two countries share an identical vaccination rate, disparities may persist in terms of the pace at which their respective populations attain vaccination coverage.

The stepwise regression model, a widely utilized approach in statistical analysis, has been applied in our study for variable selection in regression models. There are three primary reasons behind the selection of this method:

(1) Efficient Variable Selection: Stepwise regression enables an efficient and systematic process of selecting variables that significantly impact the outcome of interest. By iteratively evaluating and including or excluding predictors based on their statistical significance, this method allows us to focus on the most influential variables while reducing computational complexity.

(2) Model Parsimony: A key stepwise regression objective is creating a parsimonious model. By including only the most relevant variables, we can achieve a balance between model complexity and interpretability. This approach helps prevent overfitting and ensures the resulting model is more easily understood and generalizable.

(3) Objective and Automated Approach: Stepwise regression provides an objective and automated approach to variable selection. It prevents us from making subjective decisions about which variables to include or exclude. Following a systematic algorithm based on statistical significance helps ensure consistency and standardization in the variable selection process.

## 4. Survival Analysis Results

**Figure 1:**
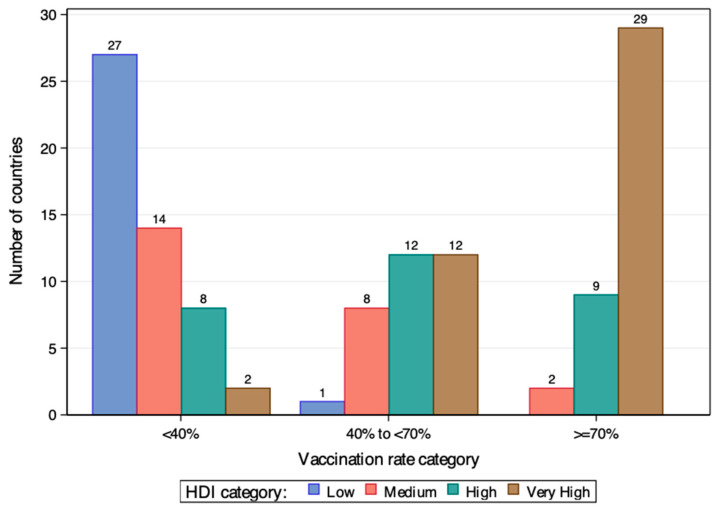
Country vaccination rates at the end of 2021 by HDI category.

**Figure 2:**
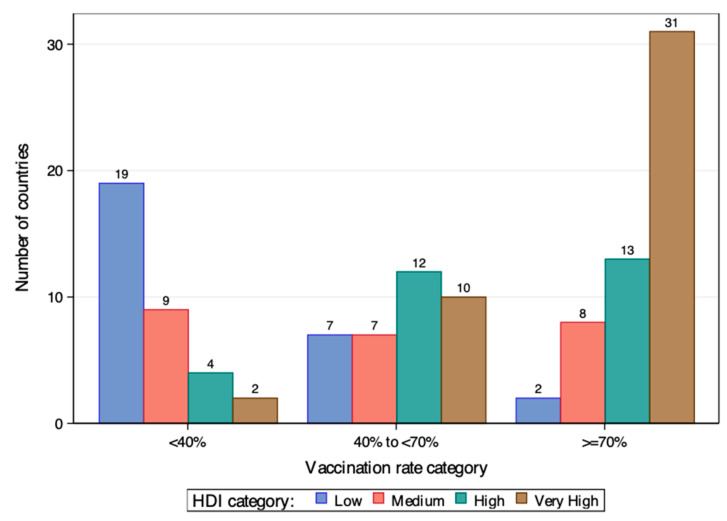
Country vaccination rates at the end of 2022 by HDI category.

Figure 1 and Figure 2 show the country’s vaccination rate by HDI category at the end of 2021 and 2022 among the 124 countries, respectively. By end of 2021, only one low HDI country had a vaccination rate over 40%, while the rest were all in the lowest rate group. In contrast, countries of high and very high HDI dominate the ≥ 0% rate group. Among the 40 countries in this group, 38 were from the high and very high HDI group. Situations improved by end of 2022 for low and medium HDI countries, but the inequity was still there. While some of them caught up to the pace of developed countries, they still comprised the majority of the <40% rate group.

**Figure 3:**
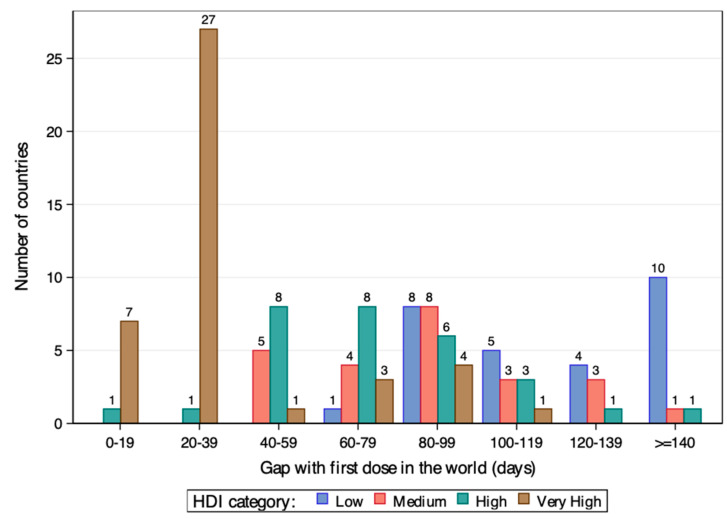
Countries’ first COVID-19 vaccination by HDI and gap category.

**Figure 4:**
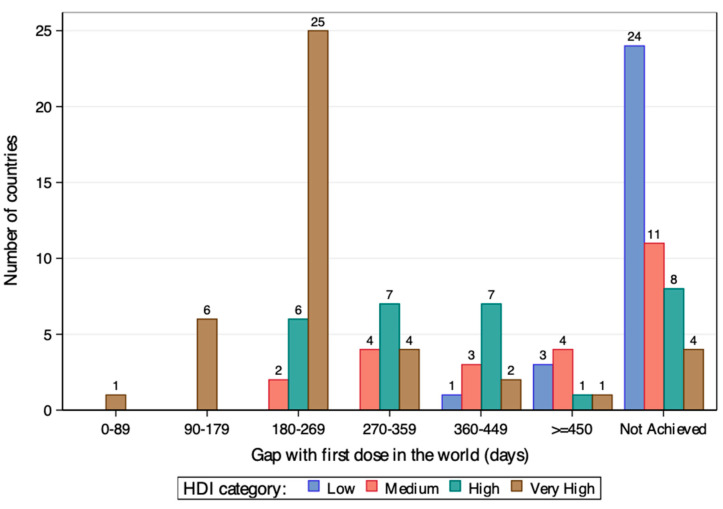
Countries achieving 50% COVID-19 vaccination rate at the end of 2022 by HDI and gap category.

This inequity can also be found in a longitudinal way. Figure 3 shows how long it took for countries to access the first COVID-19 vaccination by HDI category. While in the first 39 days, the vaccine was available for most very high HDI countries (34 of 43), it took more than 80 days for the majority of the very low HDI countries to receive the first dose of vaccination. In Figure 4, we evaluate the time from world first COVID-19 vaccination dose to 50% population vaccinated of each country in days. We can see from the figure that in the first half-year, seven very high HDI countries achieved this goal. While it took almost 1 year for the first low HDI country to achieve the same. What is more serious is that there were still 47 countries of the 124 that had not achieved the 50% vaccination rate by the end of 2022, and about 75% of them were low or medium HDI countries.

Table 3 presents the results of the Cox regression model. The following variables were determined to be significant based on the stepwise selection process: population, population density, human development index, gender inequality index, and hospital beds per thousand people. These variables demonstrated a statistically significant relationship with the event being studied. Conversely, the remaining variables were unrelated to the event, as they did not meet the significance criteria during the stepwise selection process. Therefore, they were not included in the final model. First, countries with larger populations and higher population densities had a greater probability of achieving a 50% vaccination rate. Controlling for other factors, an increase in population of 10 million was associated with a 1.2% increase in the probability of achieving the target rate ([HR] = 1.012, 95% CI 1.002–1.022). Similarly, for every 100 per km^2^ increase in population density, the probability increased by 13.3% ([HR] = 1.133, 95% CI 1.008–1.273). Second, there was a positive association between the human development index (HDI) and the probability of achieving the target rate. A 0.01 increase in HDI was associated with an 8.4% increase in the probability ([HR] = 1.084, 95% CI 1.027–1.143). Third, countries with higher gender inequality index exhibited a lower probability of achieving the target rate. For every 0.01 increase in the index, there was a 5.3% decrease in the probability of achieving the target rate ([HR] = 0.947, 95% CI 0.910–0.986). Fourth, countries with a higher number of hospital beds per population had a lower probability of achieving a 50% vaccination rate. Increasing the number of hospital beds by one per 1000 people was associated with a 28.7% decrease in the probability ([HR] = 0.713, 95% CI 0.616–0.825).

With regard to the missing data, in our final model, only three independent variables had missing values. The table below (Table 4) shows details of the missing data. We further summarized the dependent variables by missing and non-missing groups according to available data.

According to the information from the above table, we can see that there were few values missing. Also, we do not see a big difference in the dependent variables between the missing and non-missing groups. So, we used the average value to impute the missing values and re-ran the final model to see if there was any bias (see Table 5).

The re-run result is displayed above. Compared with our previous results, it shows that population density has a *p*-value larger than 0.05. We used both population and population density in our model; these two variables usually have a high correlation. Therefore, the non-significance of population density may be due to a confounding bias led by population. Overall, we believe that model results were not severely biased by the missing data, as all other variables remained significant after imputation and all risk ratios were only slightly changed. In addition, the directions of relationships were not reversed, including population density.

## 5. Discussion

National economics and the education system played a substantial role in shaping health outcomes and public health capacity, particularly during the pandemic. Our study findings indicate that countries with low Human Development Indexes experienced the longest time initiating their first vaccine rollout, with extended durations observed in the upper range. The inequitable distribution of COVID-19 vaccines is often attributed to vaccine hesitancy or barriers to uptake in low and medium HDI countries. We anticipate that this delayed vaccine access would disproportionately impact COVID-19 outcomes in these countries, leading to more negative consequences. In addition to the development level, the population also played an important role in our study’s vaccination access. We found that countries with a larger population and higher population density were more likely to vaccinate their people earlier. This may be because world vaccination resources were more likely to be provided to countries with larger populations. We also found that gender inequality showed a positive relationship with vaccination inequality. According to our study, people from countries with larger gaps between genders had more difficulties in vaccinating their populations. This indicated that gender inequality might also exist in COVID-19 vaccination. In other words, it seems that men are more likely to receive the vaccination than women, especially in countries with larger gender gaps. 

### 5.1. Implications for Global COVID-19 Vaccination Strategies

These findings highlight the importance of prioritizing equitable, timely, and appropriate access to COVID-19 vaccines in countries with the smallest economies. By focusing on the practical implications and real-world consequences of these delays, our research adds meaningful context to the existing literature in this field [24,25,26,27,28,29]. Furthermore, this research contributes to the broader literature on health equity and the social determinants of health. It highlights the importance of addressing the systemic factors contributing to health disparities, emphasizing the need for comprehensive, multi-level interventions to achieve equitable health outcomes.

In this study, we investigated the timing of vaccine access at the country level and examined how delays were influenced continuously by a country’s economic status. By analyzing data on vaccination rates and economic and health system indicators, we aimed to identify any associations, or modifications thereof, between a country’s economic status and the duration of delays in accessing vaccines. This research provides insights into the potential impact of economic, health, and educational factors on vaccine distribution and highlights the importance of addressing disparities in access based on economic standing.

### 5.2. Limitations and Future Directions

Firstly, this study primarily focused on assessing vaccination inequity at the country level by examining vaccination rates, without considering the variations in vaccine producers and the quality of vaccines administered. Consequently, the study could not discern inequalities arising from differences in access to superior vaccines among countries. Secondly, the study utilized publicly available online data from five dimensions, namely population, education, health resources, development level, and COVID-19 epidemic severity. However, certain factors, particularly the attitudes and perceptions of individuals within each country, proved challenging to quantify and collect through online sources. For instance, factors such as public awareness and acceptance of vaccines may significantly influence vaccination rates but were not adequately captured by available online data at the country level. These factors may only be effectively assessed through targeted surveys administered to representative samples of the population. 

Furthermore, studies examining public attitudes toward vaccines have demonstrated that vaccine acceptance and hesitancy can vary across populations and countries. Factors such as vaccine confidence, trust in healthcare systems and authorities, misinformation, and social influences can significantly impact vaccine uptake [25,30,31,32]. These factors were not adequately captured in the current study due to limitations of the available online data. Thus, further research is warranted to explore the underlying reasons for vaccination inequality by conducting population-based surveys across different countries. To reach further research frontiers about vaccine equity, it is crucial to prioritize efforts to identify each country’s specific requirements in terms of data tracking, surveillance, and timely reporting. 

## 6. Conclusions

In concordance with the extant literature, our observations and findings provide valuable insights into the inequality in COVID-19 vaccination rates among countries. The use of a Cox regression model allowed for a comprehensive analysis of various factors that influence the speed at which countries achieve 50% population vaccination. The study identified several significant predictors, including population size, population density, Human Development Index, Gender Inequality Index, and hospital beds per 1000 people.

The results indicate that countries with larger populations and higher population densities tend to achieve 50% population vaccination at a faster rate. This can be attributed to the availability of more resources and infrastructure to administer vaccines efficiently to a larger number of people. Additionally, countries with higher Human Development Index scores, which reflect better socioeconomic conditions and healthcare systems, also showed faster progress in vaccination. The study also highlights the impact of gender inequality on vaccination rates. Countries with lower Gender Inequality Index scores, indicating better gender equality, demonstrated a higher likelihood of achieving vaccination goals quickly. This suggests that addressing gender disparities and promoting equal access to vaccines can contribute to more equitable vaccination rates. The analysis also considered the impact of development level on vaccination outcomes. 

The paper highlights the importance of addressing structural impediments to vaccination awareness, access, and uptake. It emphasizes that healthcare professionals, scholars, and policymakers should identify and overcome barriers to immunization to ensure future vaccination campaigns are not hindered by these challenges. This could involve targeted interventions to reach vulnerable populations, building trust, improving healthcare infrastructure, and fostering international collaboration.

Overall, this study provides valuable insights into the factors influencing COVID-19 vaccination rates and highlights the need for global efforts to address vaccine inequity. By understanding the predictors of vaccination rates and the impact of development levels, policymakers can make informed decisions and implement strategies to ensure inclusive and equitable vaccine access. Addressing the needs and strategies for resilience building will inherently vary across different countries and regions, reflecting their unique contextual factors. Prioritization of vulnerable populations in vaccine distribution strategies is crucial, with targeted initiatives to reach marginalized communities and ensure equitable vaccine access. Moreover, the donation and sharing of surplus vaccine supplies by HICs to LMICs, facilitated by international collaborations like the COVAX initiative, are essential steps toward global health equity. Supporting local vaccine production in LMICs can also bolster self-reliance, decrease external dependencies, and advance equitable vaccine access through technology transfer, skill development, and investment in manufacturing infrastructure. 

In summation, the significance of this research extends to its capacity to inform policymaking and intervention strategies aimed at diminishing socioeconomic and health-related disparities in immunization rates, thus enhancing health equity. While the study did not directly investigate racial and ethnic disparities in COVID-19 vaccination rates, it indirectly acknowledged the presence of structural inequities that can contribute to disparities in vaccine uptake among populations of low socioeconomic status. The COVID-19 pandemic has imposed unparalleled tribulations upon individuals, communities, and sovereign entities, affecting health, economic stability, education, and general welfare. Addressing the pandemic’s effects from an equity lens with a statistical approach underscores the necessity of collective action, resilience, and innovation in confronting such global health emergencies.

## Figures and Tables

**Table 1:** Source of the used data.

Data	Source	Detail
Vaccination rate	https://github.com/owid/covid-19-data/blob/master/public/data/vaccinations/vaccinations.csv (accessed on 1 July 2023)	updates daily by the Our World in Data team from official reports
Population	https://github.com/owid/covid-19-data/blob/master/public/data/owid-covid-data.csv (accessed on 1 July 2023)	United Nations, Department of Economic and Social Affairs, Population Division, World Population Prospects, 2019 Revision
Population density	https://github.com/owid/covid-19-data/blob/master/public/data/owid-covid-data.csv (accessed on 1 July 2023)	World Bank World Development Indicators, sourced from Food and Agriculture Organization and World Bank estimates
Median age	https://github.com/owid/covid-19-data/blob/master/public/data/owid-covid-data.csv (accessed on 1 July 2023)	UN Population Division, World Population Prospects, 2017 Revision
Human development index	https://github.com/owid/covid-19-data/blob/master/public/data/owid-covid-data.csv (accessed on 1 July 2023)	United Nations Development Programme (UNDP); range from 0 to 1, with higher index meaning better development
GDP per capita	https://github.com/owid/covid-19-data/blob/master/public/data/owid-covid-data.csv (accessed on 1 July 2023)	World Bank World Development Indicators, sourced from World Bank, International Comparison Program database
Gender inequality index	https://ourworldindata.org/grapher/gender-inequality-index-from-the-human-development-report?tab=chart (accessed on 1 July 2023)	UNDP, Human Development Report (2021–22) (2022); range from 0 to 1, with higher index meaning larger inequality between two genders
Healthcare access and quality index	https://ourworldindata.org/grapher/healthcare-access-and-quality-index (accessed on 1 July 2023)	Institute for Health Metrics and Evaluation (2017), World Health Organization (via World Bank); range from 0 to 100, with higher index meaning better healthcare resources.
Hospital beds	https://github.com/owid/covid-19-data/blob/master/public/data/owid-covid-data.csv (accessed on 1 July 2023)	OECD, Eurostat, World Bank, national government records, and other sources
Completion rate of primary education	https://ourworldindata.org/grapher/completion-rate-of-primary-education (accessed on 1 July 2023)	UNESCO Institute for Statistics (UIS)
Infection cases of COVID-19	https://github.com/owid/covid-19-data/blob/master/public/data/owid-covid-data.csv (accessed on 1 July 2023)	Updated daily based on COVID-19 Dashboard by the WHO
Mortality due to COVID-19	https://github.com/owid/covid-19-data/blob/master/public/data/owid-covid-data.csv (accessed on 1 July 2023)	Updated daily based on COVID-19 Dashboard by the WHO

**Table 2:** Description of characteristics of the sampled countries.

Category	Statistics
Population (million)	
N	124
mean (sd)	63.226 (183.3235)
median (min, max)	19.838 (5.023, 1425.887)
Population density (per km^2^)	
N	121
mean (sd)	256.050 (957.7058)
median (min, max)	82.328 (3.202, 7915.731)
Median age (years)	
n	124
mean (sd)	29.835 (9.7002)
median (min, max)	29.000 (15.100, 48.200)
Human development index	
n	124
mean (sd)	0.711 (0.1668)
median (min, max)	0.722 (0.361, 0.957)
Human development index category	
Low	28 (22.6)
Medium	24 (19.4)
High	29 (23.4)
Very High	43 (34.7)
GDP per capita (USD)	
n	120
mean (sd)	17,555.24 (18,249.97)
median (min, max)	11,019.02 (661.240, 85,535.38)
Gender inequality index	
n	120
mean (sd)	0.357 (0.2086)
median (min, max)	0.398 (0.013, 0.820)
Healthcare access and quality index	
n	123
mean (sd)	63.250 (17.3428)
median (min, max)	63.700 (28.600, 91.800)
Hospital beds (per thousand)	
n	110
mean (sd)	2.855 (2.6418)
median (min, max)	1.950 (0.100, 13.050)
Completion rate of primary education (%)	
n	116
mean (sd)	83.664 (20.6688)
median (min, max)	96.000 (29.000, 100.000)
Infection cases of COVID-19 by the end of 2022 (per thousand)	
n	121
mean (sd)	131.783 (167.5818)
median (min, max)	58.038 (0.354, 637.706)
Death rate due to COVID-19 by the end of 2022 (per thousand)	
n	121
mean (sd)	1.143 (1.3479)
median (min, max)	0.576 (0.001, 6.501)

**Table 3:** Hazard ratios of selected variables in the survival model.

Descriptive	Hazard Ratios (95% CI)	*p* Value
Population increased by 10 million	1.012 (1.002, 1.022)	0.0152
Population density increased by 100 per km^2^	1.133 (1.008, 1.273)	0.0363
Human development index increased by 0.01	1.084 (1.027, 1.143)	0.0031
Gender inequality index increased by 0.01	0.947 (0.910, 0.986)	0.0077
Hospital beds increased by 1 per 1000 people	0.713 (0.616, 0.825)	<0.0001

**Table 4:** Dependent variables by missing and non-missing group for those missing data.

Group	Counts	Events	Time to Event, Mean (SD)
Missing “population density”	3	1	604.7 (265.58)
Non-missing “population density”	121	76	464.3 (253.21)
Missing “gender inequality index”	4	2	499.8 (257.75)
Non-missing “gender inequality index”	120	75	466.7 (236.11)
Missing “hospital beds”	14	4	645.6 (187.08)
Non-missing “hospital beds”	110	73	445.1 (232.32)

**Table 5:** Final model to see if there is any bias.

Description	Hazard Ratios (95% CI)	*p* Value
Population increased by 10 million	1.015 (1.006, 1.024)	0.0013
Population density increased by 100 per km^2^	1.004 (0.985, 1.023)	0.6825
Human development index increased by 0.01	1.071 (1.031, 1.114)	<0.0001
Gender inequality index increased by 0.01	0.947 (0.918, 0.976)	0.0005
Hospital beds increased by 1 per 1000 people	0.736 (0.644, 0.842)	0.0004

## Data Availability

The data presented in this study are available on request from the corresponding author.
